# Prevalence, Types, and Outcomes of Cerebral Palsy at a Tertiary Center in Jeddah, Saudi Arabia

**DOI:** 10.7759/cureus.27716

**Published:** 2022-08-05

**Authors:** Basma A Al-Jabri, Alia S Al-Amri, Abdulkarim A Jawhari, Raghad M Sait, Reham Y Talb

**Affiliations:** 1 Pediatrics/Developmental Behavioral Pediatrics, Faculty of Medicine, King Abdulaziz University, Jeddah, SAU; 2 Pediatrics, Faculty of Medicine, King Abdulaziz University, Jeddah, SAU

**Keywords:** spasticity, epilepsy, developmental delay, gastrostomy feeding, cerebral palsy

## Abstract

Background: In developed countries, cerebral palsy (CP) is the most common neurological disorder in children. It is defined as a non-progressive disturbance to the developing brain leading to motor impairment that affects the child’s activity. CP is classified into three main subtypes: ataxic, spastic, and mixed. Objectives: This study aimed to estimate the prevalence of CP and its subtypes in a single tertiary center located in Jeddah, Saudi Arabia. Method: This retrospective record review study included 98 patients diagnosed with CP from 2004 to 2019. Data were extracted from the hospital medical record and assessed using various tools. Result: The total number of patients was 98, with an estimated CP prevalence of 1.6 per 1000 lives. Most of the patients (74.8%) had spastic CP subtype, and 54.8% had quadriplegia. The mean age of the live children was 7.45 ± 3.76 years. Moreover, gastrostomy was the most favorable feeding method. Conclusion: The prevalence of CP is almost equivalent to the national and worldwide figures. Spastic CP has the highest rates. Furthermore, the male gender has been identified as a significant risk factor for CP in the local community.

## Introduction

Cerebral palsy (CP) is the most common neurological disorder leading to physical disability in children in developed countries [1]. It is defined as non-progressive disturbances to the developing brain leading to motor impairment that affects the individuals' activities throughout their life [2]. The etiology related to CP varies widely, but the majority is related to the perinatal period [1]. Furthermore, CP is classified clinically into motor sub-types, including ataxic, spastic (unilateral and bilateral), dyskinetic (choreoathetotic, dystonic), and mixed [3]. Such a classification depends on the affected area in the central nervous system [4]. Nowadays, several studies have estimated that the overall prevalence of CP has decreased worldwide [3, 5-9]. It is estimated to affect three to four individuals out of 1000 individuals in the general population [5]. Economically, this disorder costs the United States about 11.5 billion dollars for each affected infant [10].

The motor disability of CP patients is usually associated with different impairments, such as communication problems mainly expressive language disorder, behavioral disturbances, sensory impairment, seizure disorders, and intellectual disability [11]. Neonatal seizures are the most common clinical manifestation of congenital or acquired abnormalities in the brain of newborns [12]; according to reports, 10% of children with epilepsy and nearly one-third of children with CP have a history of neonatal seizures [13]. Other significant issues affecting CP patients are feeding difficulties secondary to oral motor dysfunction [14]. They are common among children with bilateral dyskinetic or bilateral spastic CP involving four limbs [14-16]. Such cases have high morbidity due to the increased risk of aspiration pneumonia and malnutrition [16].

A systemic review which was conducted in 2013, comprising 49 studies, proposed that the prevalence of CP had remained constant in recent years regardless of the increase in the survival rate of at-risk preterm babies [10]. In other words, it suggested other possible risk factors that may play a significant role in assessing the incidence and the severity of CP and that needed to be reviewed.

In the Kingdom of Saudi Arabia (KSA), there are limited studies about the prevalence of CP across the kingdom's major regions and cities. A study done in 2006 revealed that the prevalence of CP in a single tertiary hospital in Riyadh was 4.1 per 10,000, which is relatively high [17]. However, Al Salloum et al. reported a lower prevalence of 2.3 per 1000 live birth prevalence of CP [18].

This study aimed to assess the prevalence, the subtypes, and the associated outcomes with CP in a tertiary center in the Western region of Saudi Arabia. 

## Materials and methods

Patient selection

This study comprised a hospital-based, retrospective record review of children referred to the pediatric clinic at King Abdulaziz University Hospital (KAUH), Jeddah, Saudi Arabia, between January 2004 and January 2019. CP patients and their maternal variables during pregnancy were included in this cohort study. This study was approved by the Research and Ethics Committee of the hospital.

Subjects

The population included all the patients confirmed to have CP at two years old and follow up in KAUH. The diagnosis was made by consultants of either Developmental Pediatrics or Child Neurology. The prevalence of CP children was calculated by dividing the total number of identified cases in this center which was 98 cases by the total number of children born during the same period in the same center which was 61228 births. 

Data extraction

Subtypes

The CP motor subtypes were described as follows: (1) Spastic diplegia: primarily compromises both of the lower limbs, where there might be upper limbs involvement, but the tone abnormality must be less than that for the lower limbs. (2) Spastic triplegia: primarily involves three limbs; both of the lower limbs in addition to one upper limb, where the lower limb is dominantly spastic, or both of the upper limbs and one lower limb, or upper limb dominantly spastic. (3) Spastic quadriplegia: it involves all four limbs and double hemiplegia, in which the upper limbs are more affected than the legs. (4) Spastic hemiplegia: indicates a single hemi-body part, including the involvement of only one limb (monoplegia). (5) Dyskinetic: includes a combination of dystonia or choreoasetothic movements. (6) Ataxic: a failure in muscular coordination resulting in abnormal accuracy, force, or rhythm [19-20].

Associated Symptoms 

Epilepsy

Epilepsy was described as two unprovoked seizures, omitting neonatal or febrile seizures. Types of epilepsy were documented at the time of clinical visit or admission. The use of antiseizure medications was also recorded.

Feeding

Based on feeding behavior, the patients were classified as independent (0), need some assistance (1), totally dependent on an assistant (2), partly tube fed (3), and mainly tube fed (4). In addition, the presence of gastrostomy was documented.

Risk Factors

Maternal data at the time of the patient pregnancy and obstetric variables were obtained from the patients’ records [6].

Data Analysis

For data entry and data analysis, we used the Statistical Package for Social Sciences version 21 (SPSS v.21, IBM Corp., Armonk, NY). Regrouping of variables was done based on the study objectives. Categorical variables were represented as frequencies and percentages, while quantitative variables were represented as mean and standard deviation. The prevalence was depicted as a percentage with a 95% confidence level. 

## Results

Prevalence and demographics

Between 2004 and 2019, 98 children born were diagnosed with CP leading to an overall prevalence of 1.6 per 1000 live births. As shown in Table 1, the mean age of the live children was 7.45 ± 3.76 years. About 59.2% of children were males, 52% had Saudi nationality, with a mean birth weight of 2.87 ± 0.97 kg. 

**Table 1:** Demographic and clinical characteristics among studied patients.

Demographic			Patients	
Age of participant (year)				
Live (Mean ± SD)			7.45±3.76	
Deceased (Mean ± SD)			5.29±5.29	
Gender				
Male; n (%)			58 (59.2%)	
Female; n (%)			40 (40.8%)	
Nationality				
Saudi; n (%)			51 (52%)	
Non-Saudi; n (%)			47 (48%)	
Birthweight (kg)				
Mean ± SD			2.87± 0.9	
Median			2.8	
Occurrence of CP congenital (fetal and ≤28 days of life)			57	
Post-neonatal (final diagnosis of CP after 28 days and before two years of life)			41	

The etiology of the CP was congenital in 37 (37.8%) of the participants, post-natal in 18 (18.4%), and undetermined in 43 (43.9%) participants (Table 1).

Subtypes of CP

Of the 98 children, 73 (74.5%) had the spastic CP subtype, one (1%) had dyskinetic CP subtype, and the type was not determined in 24 (24.5%) participants. Of the 73 (74.5%) participants with spasticity, 40 (54.8%) had bilateral quadriplegia, 14 (19.2%) had bilateral diplegia, four (5.5%) had unilateral right hemipelagic, one (1.4%) had unilateral left hemialgia, and 14 (19.2%) had unspecified spasticity.

Feeding Behavior, Epilepsy Status, and Development of CP Patients

As shown in Table 2, 44.9% of the participants were fed via gastrostomy, 68.1% were actively seizing, and 60.2% were developmentally delayed.

**Table 2:** Associated complications with CP. NGT, nasogastric tube; CP, cerebral palsy

Associated complications		CP subtypes no (%)	Spastic quadriplegic	Spastic diplegic	Unilateral hemiplegic (right-left)	Dyskinetic	Unspecified spasticity	Unspecified
									Feeding	Gastrostomy		23 (23.5%)	5 (5.1%)	1 (1%)-0 (0)	0 (0)	7 (7.1%)	8 (8.2%)
										Oral		7 (7.1%)	5 (5.1%)	3 (3.1%)-1 (1%)	0 (0)	5 (5.1%)	9 (9.2%)
	NGT		10 (10.2%)	4 (4.1%)	0 (0)-0 (0)	1 (1%)	2 (2%)	7 (7.1%)
Epilepsy	Generalized tonic-clonic		38 (38.8%)	6 (6.1%)	1 (1%)-0 (0)	0 (0)	10 (10.2%)	12 (12.2%)
									Developmental delay			29 (29.6%)	5 (5.1%)	3 (3.1%)-1 (1%)	1 (1%)	8 (8.2%)	12 (12.2%)
Total number of patients: 98																	

## Discussion

This study was carried out at King Abdulaziz University Hospital (KAUH) to determine the prevalence, the etiology of CP, and its subtypes. The neonatal survival rates of born infants from 2005 to 2019 in the University hospital-based cohort study were high. However, the prevalence of CP has shown a slight increase during that specific period, with an estimated prevalence of 1.6 per 1000 live birth in comparison to the study conducted by Ansari and Akhdar (1997), which showed a prevalence of 1.2 per 1000 live birth [21]. This finding indicates improvement in CP diagnosis, owing to advancements in diagnostic tools for CP such as CT scan of the brain, MRI of the brain, and electroencephalogram (EEG) [21]. Another study conducted in the Asir region showed CP as the second most common neurological disorder with a prevalence of 18.9%, which corroborated our findings regarding the increase in CP diagnostic rate [22]. However, a recent study conducted in Riyadh has estimated a lower prevalence of 0.41 per 1000 live born, which may be attributed to data collection from a single center [23]. This finding indicates the significance of conducting a multi-centric survey when determining the prevalence and the pattern of a disorder. It also revealed that CP prevalence varied across the different regions of Saudi Arabia. Considering its geographical location, Jeddah is one of the most cosmopolitan cities in the world. It is the principal gate to the two holy cities for Islam: Makkah and Madina. It hosts millions of Muslims around the year from all over the world on their way to Hajj. This explains having different nationalities other than Saudi seeking medical care in different hospitals in Jeddah.

Compared to international data, the prevalence of CP in this study is almost comparable. The result suggested that the prevalence rate of CP in KAUH is in the middle among worldwide figures representing the lowest rates in Netherlands and Turkey, which was estimated as 1.19 and 1.10 per 1000 live birth, respectively [24], and the highest rates in UK and USA, which were estimated as 3.90 and 3.34 per 1000 live birth, respectively [24]. The increased survival rates of at-risk preterm infants could justify the high incidence in UK and USA. Similar to our study, a Chinese research also estimated the prevalence as 1.6 per 1000 live births [25]. Moreover, several studies showed a reflecting apparent decrease in CP prevalence in comparison to their previous records data [9-10, 24]. However, their current prevalence is approximately similar to this study, adding more to the accuracy and the reflection of this study to international data. 

This study reported spastic CP as the most common type of CP and the second most common type was dyskinetic (47.5% and 1%, respectively). These findings were corroborated by a previous study in Riyadh that reported spasticity to be the most common type of CP in Saudi Arabia by (61.51%) [17]. Globally, spastic CP has been shown to be the most common type of CP across various continents, ranging from 42% to 92% [24].

Furthermore, this study showed that CP was predominantly incident in male children (male: female = 59.2%:40.8%). Previous studies conducted in Saudi Arabia reported that males are predominantly affected by CP (male: female = 62.62%:37.38%) [17]. Global data also showed CP to be more incident in males than females (58% vs. 42%) [26].

Furthermore, we observed that most of our cohort was fed through gastrostomy (44.9%), followed by orally (30.6%), and via nasogastric tube (NGT) (24.5%). Our results indicated a substantial correlation between severe disability and feeding intolerance among CP patients. Our results can be corroborated by another European study that stated children with CP have feeding disorders, commonly dysphagia, by 58%. It is secondary to the central nervous system insult, which increases the risk of aspiration and potential pulmonary complications with oral feeding [27]. These findings highlight the significance of comprehensive management of such patients and close monitoring in the long term. 

Epilepsy was reported as the most common impairment among our cohort (68.1%). A previous Saudi study also supported epilepsy as the most common impairment among CP patients [17]. This finding suggests the importance of periodic screening of such impairment and an early inference to avoid possible complications, such as intractable epilepsy and aspiration, which justifies the need for an interventional approach and strict management to reach a full recovery of seizure or complete remission and improvement in the morbidity and mortality rates of those patients [28].

Lastly, the etiology of CP was determined to be congenital or prenatal in 57% of our cohort. On the other hand, CP etiology was post-neonatal in only 41% of our patients. This finding suggested that prenatal risk factors play a significant role in the incidence of CP. In addition, a Danish study had shown that the majority of CP children have a strong association with maternal smoking, vaginal infection, and other prenatal causes that clarify the increased risk of CP children [29]. This demands good management and interventions to the prenatal risk factors by adhering to an early screening, investigation, and strict prevention protocol for lesser prevalence rates in the future.

## Conclusions

The prevalence of CP in this single tertiary center corresponds to the national and international statistics. Spasticity has the highest prevalence rate. However, a large number of population-based multicenter studies in different regions are needed to establish an understanding of the pattern of CP and major risk factors that play a role in CP progression in Saudi Arabia. Moreover, the male gender was classified as a risk factor for CP. Epilepsy has been shown to be the most common associated comorbidity. Finally, the etiology of CP mainly comprises a congenital or prenatal cause. This emphasizes the role of the clinicians in modifying maternal risk factors and in promoting better health that might lead to good outcomes with lower CP incidence. Additional projects are needed to monitor CP patients as a standard of care in local institutes in Saudi Arabia, aiming for an early diagnosis and improvement of CP outcomes.
